# Vegetable Diversity, Injurious Falls, and Fracture Risk in Older Women: A Prospective Cohort Study

**DOI:** 10.3390/nu10081081

**Published:** 2018-08-13

**Authors:** Marc Sim, Lauren C. Blekkenhorst, Joshua R. Lewis, Catherine P. Bondonno, Amanda Devine, Kun Zhu, Richard J. Woodman, Richard L. Prince, Jonathan M. Hodgson

**Affiliations:** 1 School of Medical and Health Sciences, Edith Cowan University, Joondalup, WA 6027, Australia; l.blekkenhorst@ecu.edu.au (L.C.B.); joshua.lewis@ecu.edu.au (J.R.L.); c.bondonno@ecu.edu.au (C.P.B.); a.devine@ecu.edu.au (A.D.); jonathan.hodgson@ecu.edu.au (J.M.H.); 2 Medical School, Royal Perth Hospital Unit, The University of Western Australia, Perth, WA 6000, Australia; 3 Centre for Kidney Research, Children’s Hospital at Westmead, School of Public Health, Sydney Medical School, The University of Sydney, Sydney, NSW 2145, Australia; 4 Department of Endocrinology and Diabetes, Sir Charles Gairdner Hospital, Nedlands, WA 6009, Australia; kun.zhu@uwa.edu.au (K.Z.); richard.prince@uwa.edu.au (R.L.P.); 5 Medical School, Sir Charles Gairdner Unit, The University of Western Australia, Nedlands, WA 6009, Australia; 6 Flinders Centre for Epidemiology and Biostatistics, Flinders University, Adelaide, SA 5042, Australia; richard.woodman@flinders.edu.au

**Keywords:** nutrition, epidemiology, ageing, musculoskeletal health, geriatrics, injury

## Abstract

The importance of vegetable diversity for the risk of falling and fractures is unclear. Our objective was to examine the relationship between vegetable diversity with injurious falling and fractures leading to hospitalization in a prospective cohort of older Australian women (*n* = 1429, ≥70 years). Vegetable diversity was quantified by assessing the number of different vegetables consumed daily. Vegetable intake (75 g servings/day) was estimated using a validated food frequency questionnaire at baseline (1998). Over 14.5 years, injurious falls (events = 568, 39.7%), and fractures (events = 404, 28.3%) were captured using linked health records. In multivariable-adjusted Cox regression models, women with greater vegetable diversity (per increase in one different vegetable/day) had lower relative hazards for falls (8%; *p* = 0.02) and fractures (9%; *p* = 0.03). A significant interaction between daily vegetable diversity (number/day) and total vegetable intake (75 g servings/day) was observed for falls (*p*_interaction_ = 0.03) and fractures (*p*_interaction_ < 0.001). The largest benefit of higher vegetable diversity were observed in the one third of women with the lowest vegetable intake (<2.2 servings/day; falls HR 0.83 95% CI (0.71–0.98); fractures HR 0.74 95% CI (0.62–0.89)). Increasing vegetable diversity especially in older women with low vegetable intake may be an effective way to reduce injurious fall and fracture risk.

## 1. Introduction

Healthy lifestyles which incorporate a balanced diet and regular physical activity are commonly promoted by public health organizations for their benefits on general well-being. The promotion of increased vegetable intake is a major component of global public health advice. This advice is supported by abundant evidence indicating health benefits in higher vegetable intakes, especially in the primary prevention of chronic illness [1,2]. However, vegetables contain a variety of nutrients and phytochemicals, possibly explaining why some vegetables may have larger health benefits in comparison to others [3]. Therefore, it is plausible that diets incorporating higher vegetable diversity could provide superior health benefits.

Approximately 30% of the population aged ≥65 years report a fall annually, with this increasing to 42% for those aged ≥75 years [4]. Fractures usually occur after an injurious fall and account for 4–6% of all falls [5]. Considering the link between falling and fractures [6], this represents a very large concern for older populations. Falls often do not result from a single cause, but involve multiple interactions between an individual with a propensity to fall and acute mediating factors [7]. Compromised visual, proprioceptive and vestibular systems, together with reduced neuromuscular function contribute to impaired balance recovery as part of the ageing neuromuscular system [7]. Such characteristics present a high level of complexity when attempting to reduce the risk of falls in older populations.

Higher total vegetable consumption in older populations have been inversely associated with fall propensity risk factors, such as muscle strength [8], physical function [9], and disability [10]. As such, Australian Dietary Guidelines recommend a combination of keeping physically active and consuming a range of nutritious food (including a diverse range of vegetables) to maintain muscle and bone health for older people [1]. For example, diets rich in vegetables and/or fruit have been inversely associated with sarcopenia [11]. Previously, we have also demonstrated that higher total vegetable intake is associated with lower fracture risk [12]. To date, however, the importance of vegetable diversity remains unclear. The primary aim of this investigation was to examine the associations of vegetable diversity, with 14.5 years of injurious falls and fractures (resulting in hospitalization) in a prospective cohort of postmenopausal women aged ≥70 years.

## 2. Materials and Methods

### 2.1. Study Population

The population included females in the Perth Longitudinal Study of Aging in Women (PLSAW). The women were originally recruited to a five-year, double-blind, randomized controlled trial of daily calcium supplementation to prevent fracture, the Calcium Intake Fracture Outcome Study (CAIFOS). The women were included on the basis of an expected survival beyond five years and not receiving any medication (including hormone replacement therapy) known to affect bone metabolism and has been previously described [13]. The women (*n* = 1500) were recruited from the Western Australian general population of women aged ≥70 years by mail using the electoral roll, which is a requirement of Australian citizenship. At the completion of the five-year trial, women were invited to participate in two follow-up observational studies. Total follow-up was 14.5 years. A total of 1485 women completed a food frequency questionnaire at baseline in 1998. Participants (*n* = 17/1485, 1.1%) with implausible energy intakes (<2100 kJ (500 kcal) or >14,700 kJ (3500 kcal)) or undertaking vitamin D supplementation (due to its link with falling [14], *n* = 39/1485, 2.6%) were not included in the analysis. The current study then included 1429 women. All participants provided written informed consent. Ethics approval was granted by the Human Ethics Committee of the University of Western Australia. Both studies were retrospectively registered on the Australian New Zealand Clinical Trials Registry (CAIFOS trial registration number ACTRN12615000750583 and PLSAW trial registration number ACTRN12617000640303) and complied with the Declaration of Helsinki. Human ethics approval for the use of linked data was provided by the Human Research Ethics Committee of the Western Australian Department of Health (project number 2009/24).

### 2.2. Dietary Assessment

A semi-quantitative food frequency questionnaire (FFQ) developed and validated by the Cancer Council of Victoria was used to assess dietary intake on a single occasion at baseline (1998) [15,16]. The FFQ instructed participants to answer all questions in regards to their usual eating habits over the past 12 months. Participants were supported by a research assistant when completing the FFQ. Vegetable diversity (number of different vegetables consumed per day; number/day) was obtained from the FFQ data using the question ‘How many different vegetables do you usually eat per day?’ Responses ranged from <1 different vegetable per day to ≥6 different vegetables per day and were coded from 0 to 6. Despite the question’s specific reference to daily vegetable diversity, answers likely reflect the typical eating habits of participants. Vegetable diversity was then further categorized into a discrete variable (≤3 number/day, 4 number/day, and ≥5 number/day) based on an approximately equal number of women in each of the three response categories. The diet assessment analysis also included intake estimates of total vegetable (number of 75 g serves consumed per day; servings/day), protein (g/day), calcium (g/day), and alcohol intakes (g/day).

### 2.3. Injurious Falls and Fracture Outcome Assessment

Fall and fracture-related hospitalizations outcomes over 14.5 years were tracked through the Western Australian Data Linkage System (Department of Health Western Australia, East Perth, Australia) and retrieved from the Western Australia Hospital Morbidity Data System (HMDS). Records were obtained for each of the study participants from 1998 until 2013 using the International Classification of External Causes of Injury codes and the International Classification of Diseases (ICD) coded diagnosis data pertaining to all public and private inpatient admissions in Western Australia. This allows ascertainment of hospitalizations independent of self-report and avoids the problems of patient self-reporting and loss to follow-up. Falls from standing height or less, not resulting from external force were included (ICD-10 codes): W01, W05–W08, W10, W18, and W19. A fall was considered injurious if it required hospitalization. Codes used for the identification of fractures included S02–S92, M80, T02, T08, T10, T12, and T14.2. Fractures of the face (S02.2–S02.6), fingers (S62.5–S62.7), and toes (S92.4–S92.5), and fractures caused by motor vehicle injuries were excluded (external cause of injury codes V00–V99). Self-reported falls were also assessed by asking participants if they experienced a fall in the three months prior to their baseline clinical visit.

### 2.4. Baseline Characteristic Assessment

Questionnaires completed at baseline were used to assess values for potential confounding variables, including age, physical activity, and smoking history. Participants were asked about participation in sport, recreation, and/or regular physical activities undertaken in the three months prior to their baseline visit [17]. The level of activity, expressed in kilojoules per day, was then calculated using a validated method applying the type of activity, time engaged in the activity, and the participant’s body weight [18]. Smoking history was coded as non-smoker or ex-smoker/current smoker if they had consumed >1 cigarette per day for more than three months at any time in their life. Body weight was measured using digital scales to the nearest 0.1 kg and height was assessed using a wall-mounted stadiometer to the nearest 0.1 cm, both whilst participants were wearing light clothes and without socks and shoes. Body mass index (BMI) (kg/m^2^) was then calculated. Treatment (placebo or calcium) over the five years of the CAIFOS trial was included as a covariate. Current medication use at baseline was used to assess prevalent diabetes mellitus. Medications were verified by participants’ general practitioner where possible and were coded (T89001–T90009) using the International Classification of Primary Care-Plus (ICPC-Plus) method which allows aggregation of different terms for similar pathologic entities as defined by the ICD-10 coding system [19]. Socioeconomic status (SES) was calculated using the Socioeconomic Indexes for Areas developed by the Australian Bureau of Statistics which ranked residential postcodes according to relative socio-economic advantage and disadvantage. Participants were then coded into six groups from the top 10% most highly disadvantaged to the top 10% least disadvantaged.

### 2.5. Statistical Analysis

Baseline characteristics were tested for differences across categories using one-way analysis of variance (ANOVA) for normally distributed continuous variables, the Kruskal-Wallis test for non-normally distributed variables, and the Chi-squared test for categorical variables. Descriptive statistics for normally distributed continuous variables were expressed as the mean ± standard deviation (SD). Non-normally distributed continuous variables were expressed as the median and interquartile range. Categorical variables were expressed as the number and proportion (%).

The primary outcomes of the study were injurious falls and any fracture that resulted in hospitalization. The primary exposure variable was the diversity of vegetables consumed daily. Follow-up was available for all women that remained in Western Australia. The follow-up period for participants commenced from their baseline visit date until the first injurious fall, fracture, or loss to follow-up due to death or the end of the study follow-up period (14.5 years). Kaplan-Meier curves and the log-rank test were used to assess unilabiate associations of vegetable diversity with injurious falls and fractures. Cox proportional hazards modelling was used to estimate the hazard ratios (HR) for vegetable diversity and injurious falls and fractures. Two models of adjustment were adopted, including age-adjusted and multivariable-adjusted. The multivariable-adjusted model included age, BMI, treatment code (calcium or placebo), prevalent diabetes mellitus, SES, physical activity, ever smoked, and intakes of energy, protein, calcium, and alcohol. Vegetable diversity (number/day) was further explored by separating the variable into three groups (≤3 number/day, 4 number/day, and ≥5 number/day) and entered as a categorical variable. We tested for a trend using the median number of vegetable diversity within each group in separate Cox proportional hazards models. Cox proportional hazards assumptions were tested using log-log plots, which were shown to be parallel indicating that proportional hazards assumptions were not violated. For the primary analysis, we treated deaths as censored. This cause-specific approach meant that the hazard ratios could be interpreted as the relative risk for an injurious fall or fracture within the follow-up period assuming an individual remains alive. This approach assumes that the risk of a fall or fracture would have remained the same during the remainder of the follow-up period in those that died as in those that did not. Statistical analysis was performed using IBM SPSS Statistics for Windows, version 24.0 (IBM Corp., Armonk, NY, USA) and Stata software, version 14 (StataCorp LLC, College Station, Texas, USA). Statistical significance was set at a two-sided type-one error rate of *p* < 0.05 for all tests.

### 2.6. Additional Analysis

The relationship between vegetable diversity (number/day) and total vegetable intake (75 g servings/day) was investigated using Spearman rank order correlation (rho). We also explored the combined effect of vegetable diversity (number/day) and vegetable consumption (75 g servings/day) on injurious falls and fractures by testing for an interaction. The nature of interaction was further explored by using age-adjusted Cox proportional hazards models to assess the relative hazard of falls and fractures related to vegetable diversity (number/day) and vegetable intake (75 g servings/day) across three equal groups of vegetable intake (low: <2.2 servings/day; moderate: 2.2–2.9 servings/day; high: ≥2.9 servings/day) or the aforementioned vegetable diversity groups. We examined the cross-sectional association between vegetable diversity and self-reported falls using logistic regression. Finally, Cox proportional hazards modelling was used to assess the associations between vegetable diversity and hip fractures over 14.5 years.

## 3. Results

Participant baseline characteristics according to the vegetable diversity categories are presented in Table 1. The median (IQR) for the number of different vegetables consumed per day was 4 (3–5). Significant differences (*p* < 0.05) in total vegetable, energy, and calcium intake were recorded between vegetable diversity groups.

### 3.1. Vegetable Diversity and Injurious Falls (Falls-Related Hospitalization)

**Figure 1:**
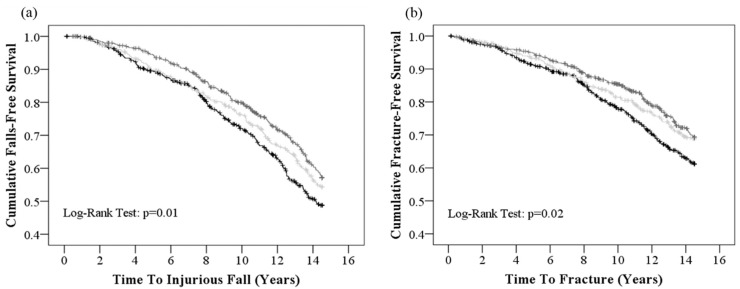
Kaplan-Meier survival curves for vegetable diversity categories for (**a**) injurious falls and (**b**) fractures over 14.5 years. Low (≤3 number/d), moderate (4 number/d), and high (≥5 number/d) vegetable diversity categories are represented by the black, light grey, and grey lines, respectively.

Over 14.5 years (15,539 person-years) of follow-up (mean ± SD; 10.9 ± 4.2 year), 39.7% (568/1429) of participants experienced an injurious fall. The number of women who experienced an injurious fall in the low (≤3 number/day), moderate (4 number/day) and high (≥5 number/day) vegetable diversity groups were 206 (42.6%), 177 (40.2%), and 185 (36.6%), respectively (Table 2). Kaplan-Meier survival curves for unadjusted injurious falls were significantly different among vegetable diversity groups (Figure 1). In the multivariable-adjusted model, increased vegetable diversity was associated with reduced hazards for an injurious fall; per increase in one different vegetable per day HR 0.92 95% CI (0.86–0.99), *p* = 0.02 (Table 2). High vegetable diversity (≥5 number/day) was associated with a 23% hazard reduction for injurious falls compared to low vegetable diversity (≤3 number/day) (Table 2).

### 3.2. Vegetable Diversity and Fracture-Related Hospitalization

A total of 28.3% (404/1429) of participants experienced a fracture over 14.5 years of follow-up (16,025 person-years). The number of women who experienced a fracture in the low (≤3 number/day), moderate (4 number/day) and high (≥5 number/day) vegetable diversity groups were 153 (37.9%), 120 (29.7%) and 131 (32.4%), respectively (Table 2). Kaplan-Meier survival curves for unadjusted fractures were significantly different among vegetable diversity groups (Figure 1). In the multivariable-adjusted model, increased vegetable diversity was associated with reduced risk for any fracture; per increase in one different vegetable per day HR 0.91 95% CI (0.84–0.99), *p* = 0.03 (Table 2). High vegetable diversity (≥5 number/day) was associated with a 22% hazard reduction in fractures compared to low vegetable diversity (≤3 number/day) (Table 2).

### 3.3. Additional Analysis

#### 3.3.1. Vegetable Diversity and Total Vegetable Intake

**Figure 2:**
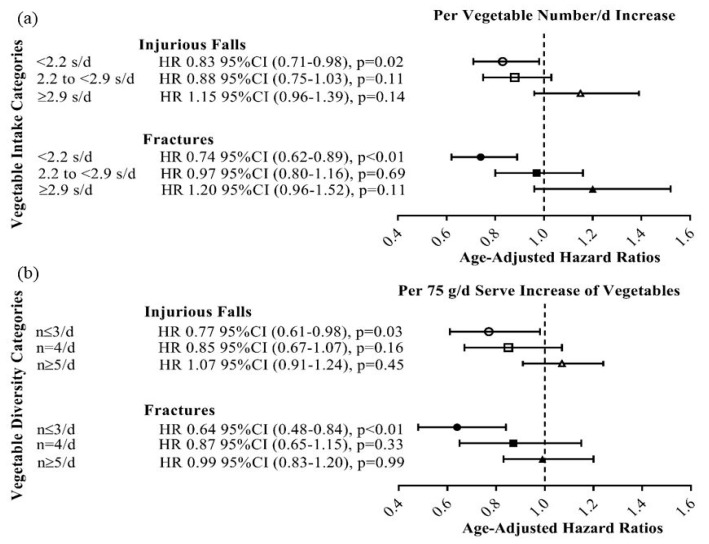
Age-adjusted hazard ratios for injurious falls and fractures based on categories of (a) vegetable intake and (b) vegetable diversity over 14.5 years. Categories for vegetable intake included: low <2.2 s/d (*n* = 476), moderate 2.2 to <2.9 s/d (*n* = 477) and high ≥2.9 s/d (*n* = 476). Categories for vegetable diversity included: low <4 *n*/d (*n* = 484), moderate 4 *n*/d (*n* = 440), and high ≥5 *n*/d (*n* = 505). Overall interaction for vegetable intake (s/d) and vegetable diversity (*n*/d) was *p*_interaction_ = 0.03 and *p*_interaction_ < 0.001 for injurious falls and fractures, respectively. *n*/d = number/day; s/d = servings/day.

Vegetable diversity (number/day) and total vegetable intake (75 g servings/day) were strongly correlated (rho = 0.77, *p* < 0.01). A significant interaction between vegetable diversity (number/day) and intake (75 g servings/day) for injurious falls (*p* = 0.03) and fractures (*p* < 0.001) was identified in the multivariable-adjusted Cox proportional hazards models. Benefits of increased diversity (number/day) or increased vegetable intake (75 g servings/day) were observed only in women with the lowest vegetable intake (<2.2 servings/day) or the lowest diversity (≤3 number/day), respectively. The age-adjusted Cox proportional hazards model demonstrated for every increase in vegetable number per day, women in the lowest category of vegetable intake (<2.2 servings/day) had a 17% and 26% lower relative hazard for an injurious fall or fracture (Figure 2). Similarly, for every 75 g serving increase in vegetable intake, individuals within the lowest group of vegetable diversity (≤3 number/day) had a 23% and 36% lower relative hazard for an injurious fall or fracture, respectively (Figure 2). A breakdown of vegetable types consumed daily (cruciferous, allium, yellow/orange/red, leafy green, legumes, and others) according to categories of total vegetable intake categories are presented in Supplementary Table S1. The percentage contribution of each vegetable type as part of the total vegetable intake for each category is presented in Supplementary Figure S1.

#### 3.3.2. Self-Reported Falls

A total of 166 women experienced a self-reported fall in the three months prior to their baseline clinical visit. Of these women, 72 (43.4%), 52 (31.3%) and 42 (25.3%) were from the low (≤3 number/day), moderate (4 number/day), and high (≥5 number/day) vegetable diversity groups, respectively. Increased vegetable diversity (number/day) was associated with lower odds for self-reported falls (per number increase per day, OR 0.82 95% CI (0.72–0.94), *p* = 0.01) in the multivariable-adjusted model. Compared to the low vegetable diversity group (≤3 number/day), women in the high (≥5 number/day) (OR 0.54 95% CI 0.36–0.82, *p* = 0.003), but not the moderate (4 number/day) OR 0.76 95% CI (0.51–1.13), *p* = 0.76 diversity groups had lower odds for a self-reported fall.

#### 3.3.3. Hip Fractures

Over 14.5 years of follow-up (17,323 person-years), 10.7% (153/1429) of women experienced a hip fracture. Hip fracture were experienced by 62 (12.8%), 42 (9.5%) and 49 (9.7%) women in the low (≤3 number/day), moderate (4 number/day), and high (≥5 number/day) vegetable diversity groups. In the multivariable-adjusted model, increased vegetable diversity was not associated with reduced hazards for hip fractures; per increase in number/d HR 0.90 95% CI (0.79–1.03), *p* = 0.13. Compared to the low vegetable diversity (≤3 number /day), moderate (4 number /day) HR 0.73 95% CI (0.49–1.10) and high (≥5 number /day) HR 0.80 95% CI (0.55–1.19) vegetable diversity was not associated with reduced hazards for a hip fracture (*p*_trend_ = 0.26).

## 4. Discussion

The health benefits of diets rich in vegetables have been studied extensively, often being inversely associated with numerous chronic illnesses [20]. However, most studies have focused on total vegetable intake, with limited work examining the importance of vegetable diversity. In this study, vegetable diversity was associated with reduced risk for injurious falls, short-term self-reported falls and fractures in a cohort of older community-dwelling Australian women. Importantly, the greatest reduction in injurious fall and fracture risk was observed with more vegetable diversity in the one third of women consuming less than 2.2 vegetable servings per day. In this group, for an increase in one different vegetable per day the hazard for injurious falls and fractures was reduced by 17% and 26%, respectively. However as these analyses were hypothesis-generating, our findings should be interpreted with caution until replicated in larger studies.

Nutritional components of vegetables differ according to type [21]. A high vegetable diversity could, therefore, be crucial in ensuring the availability of a range of nutrients and phytochemicals that play a role in healthy ageing [22]. It is for this reason that dietary guidelines of many countries promote diversity or variety, including for vegetables in the diet. Current guidelines in Australia recommend 5–6 servings of vegetables (75 g/serving) consisting of a variety of colors daily [1]. The United States dietary guidelines include the advice to “focus on variety, nutrient density, and amount”. The guidelines suggest that vegetable variety could be accomplished by choosing from all vegetable subgroups, including dark green, red and orange, legumes, starchy, and other vegetables [2]. We believe that there is scope for expanding public health messages in relation to vegetable diversity. However, there are few studies of this important topic.

The potential mechanisms through which higher diversity might beneficially influence risk of falling and fracture are uncertain. Although numerous theories on ageing exist, the negative role of free radicals (reactive oxygen species (ROS)) and potential benefits of antioxidants in the modulation of the ageing process are popular [23]. Chronic inflammation may also be another contributing factor capable of accelerating ageing [24]. Collectively, such changes may exacerbate fall propensity risk factors [25], such as the attrition of muscle strength, as well as the decline in physical and cognitive function [26,27,28]. Therefore, nutritional strategies capable of limiting oxidative stress and/or chronic inflammation should be considered.

Dietary patterns, such as the Dietary Approaches to Stop Hypertension (DASH) [29] and the Mediterranean diet [30], which include high amounts of vegetables, have been associated with reduced biomarkers of oxidative damage. Despite such findings, evidence that vegetable diversity plays an important protective role is limited. Previously, botanical diversity of plant food components within a diet has been shown to reduce oxidative biomarkers [31]. Over a short two-week period, women (mean age-48 years, *n* = 111) were allocated to either a low (LBD) or high botanical diversity (HBD) diet, designed to provide 8–10 servings of vegetables and fruit daily. Although both diets were associated with a reduction in lipid peroxidation, only the HBD diet recorded significantly lower DNA oxidation [31]. Diets rich in vegetables and fruit have also been associated with lower levels of markers for inflammation (IL-6 and C-reactive protein) and oxidative stress (F2-isoprostane) in adolescents (*n* = 285) [32]. Perhaps diets incorporating a range of vegetables delivering a variety of phytochemicals and nutrients may be capable of limiting the deleterious effects of ageing on the skeletal muscle. Consequently, better physical function may reduce fall propensity [33] and may explain the lower risk for injurious falls observed here. Of importance, the health benefits from the active components found in vegetables may extend beyond physical function, with potential to positively influence bone metabolism [34].

Bone desorption is reported to be negatively affected by oxidative stress [35,36]. For example, a strong positive association between high levels of ROS and bone loss in the development of osteoporosis has been reported in Swedish adults (*n* = 101, mean age: 55.8 years) [37]. This finding could be associated with the numerous active ingredients found in vegetables that are known to positively influence bone metabolism [34]. Specifically, within the Framingham Osteoporosis Study, higher carotenoid intake (sources include yellow, orange, red and dark leafy green vegetables) was inversely associated with four-year loss in bone mineral density (BMD) in men (*n* = 213, mean age: 75 years). The carotenoid lycopene was also associated with lower bone loss at the lumbar spine in women (*n* = 390, mean age: 75 years) [38]. Other nutrients, such as vitamin C, potassium and magnesium (found in a diverse range of vegetables) have also been associated with greater BMD and reduced bone loss at the femoral neck in premenopausal women (aged 45–55 years, *n* = 146) over 5–7 years of follow-up [39]. The aforementioned favorable effects on bone metabolism in combination with reduced risk for injurious falls (and self-reported falls, which we also observed here) may explain the lower fracture risk in our cohort.

Limitations of the study include its observational nature which: (i) increases the possibility of bias due to residual confounding; and (ii) does not permit causal links to be established. Secondly, a strong correlation was recorded between total vegetable intake and diversity, thereby making the exploration of the individual effects of diversity difficult. However, we observed a significant interaction between vegetable diversity and vegetable intake for injurious falls and fractures. These results suggest that vegetable diversity is important especially if vegetable intake is low. Alternatively, when vegetable diversity is low, increasing vegetable intake could also be beneficial. Third, despite the use of a previously validated and reproducible method of dietary intake assessment, dietary information was self-reported, which could have led to misclassification. Fourth, although we adjusted for potential confounders, such as dietary and lifestyle factors known to be associated with fall propensity and fractures, higher vegetable diversity may be a marker of a healthier lifestyle not completely captured by the lifestyle variables included as potential confounders in the multivariable-adjusted analyses. Fifth, as biomarkers associated with inflammation and/or oxidative stress were not measured, the exact mechanism through which vegetable diversity may lower injurious fall and fracture risk remains unclear. Finally, results may not be generalized to other populations, such as elderly men or younger cohorts.

Strengths of this study include the prospective design and population-based setting with ascertainment of verified injurious falls and fractures (including hip fractures) from hospital records. We also considered short-term self-reported falls, which is more commonly reported in the literature. Additionally, study participants were representative of older community-dwelling women within the Australian population [17]. Detailed information of potential confounders including diabetes, alcohol intake, socioeconomic status, physical activity, and total vegetable intake were also taken into account.

## 5. Conclusions

In summary, this investigation indicates benefits of greater vegetable diversity for reducing injurious fall and fracture risks in older community-dwelling Australian women. Of importance, the greatest benefits of increasing vegetable diversity may be observed in women with low vegetable intake. These findings could have implications for nutritional guidelines promoted by public health organizations to reduce the risk of falls and/or fractures in older community-dwelling women.

## Figures and Tables

**Table 1:** Baseline characteristics according to all participants and by vegetable diversity intake categories.

	All Participants ^1^	Vegetable Diversity ^2^		
		≤3 number/d	4 number/d	≥5 number/d
Number (%)	1429	484 (34)	440 (31)	505 (35)
**Demographics**				
Age, years	75.2 ± 2.7	75.3 ± 2.8	75.2 ± 2.7	75.0 ± 2.7
Treatment group (calcium) ^3^	716 (50.1)	240 (49.6)	222 (50.6)	254 (50.3)
Body mass index (BMI) ^4^, kg/m^2^	27.2 ± 4.8	27.3 ± 4.7	27.2 ± 4.9	27.1 ± 4.7
Smoked ever ^5^	531 (37.4)	179 (37.2)	165 (37.7)	187 (37.3)
Physical activity ^4^, kJ/day	470.6 (0.0–746.3)	451.5 (0.0–861.1)	444.3 (149.5–821.4)	865.5 (488.2–865.4)
Prevalent diabetes mellitus	90 (6.3)	36 (7.4)	22 (5.0)	32 (6.3)
**Socioeconomic status ^6^**				
Top 10% most highly disadvantaged	63 (4.4)	21 (4.4)	13 (3.0)	29 (5.8)
Highly disadvantaged	171 (12.1)	65 (13.5)	49 (11.2)	57 (11.4)
Moderate-highly disadvantaged	229 (16.2)	81 (16.8)	69 (15.8)	79 (15.8)
Low-moderately disadvantaged	216 (15.2)	77 (16.0)	63 (14.4)	76 (15.2)
Low disadvantaged	298 (21.0)	96 (19.9)	97 (22.2)	105 (21.0)
Top 10% least disadvantaged	440 (31.1)	142 (29.5)	145 (33.3)	153 (30.7)
**Dietary intakes**				
Energy, kJ/day	7102.3 ± 2078.1	6745.5 ± 2088.7	7177.4 ± 2056.5	7378.8 ± 2041.4
Vegetable intake, g/day	196.7 ± 79.3	128.5 ± 46.8	197.5 ± 49.4	261.3 ± 70.3
Protein, g/day	79.5 ± 26.6	73.6±26.9	80.6 ± 26.6	84.4 ± 25.2
Calcium, mg/day	954.0 ± 346.8	883.6 ± 340.9	973.5 ± 339.2	1004.5 ± 348.5
Alcohol, g/day	1.8 (0.3–9.8)	1.4 (0.3–9.0)	1.9 (0.3–9.2)	2.2 (0.3–10.6)

^1^ Data presented as the mean ± SD, median (interquartile range) or number (*n*) and (%); ^2^ Vegetable diversity was assessed by number of different vegetables consumed daily (number/d); ^3^ *n* = 1428; ^4^ *n* = 1427; ^5^ *n* = 1421; ^6^ *n* = 1417. Bolded numbers indicate a significant difference (*p* < 0.05) between groups using ANOVA, Kruskal-Wallis test, and Chi-square test where appropriate.

**Table 2:** Hazard ratios (HR) for injurious falls and fractures over 14.5 years by daily vegetable diversity intake categories ^1^.

		HR per number/d Increase	*P*-Value	Vegetable diversity			
				≤3 number/d	4 number/d	≥5 number/d	*p-*Trend ^2^
Injurious Falls	Number	1429	-	484	440	505	-
	Events, *n* (%)	568 (39.7)	-	206 (42.6)	177 (40.2)	185 (36.6)	-
	Age-adjusted	0.91 (0.85–0.98)	0.01	1.00 (Referent)	0.86 (0.71–1.10)	0.76 (0.62–0.93)	0.01
	Multivariable-adjusted ^3^	0.92 (0.86–0.99)	0.02	1.00 (Referent)	0.88 (0.71–1.08)	0.77 (0.63–0.95)	0.01
Fractures	Number	1429	-	484	440	505	-
	Events, *n* (%)	404 (28.3)	-	153 (37.9)	120 (29.7)	131 (32.4)	-
	Age-adjusted	0.90 (0.83–0.97)	0.01	1.00 (Referent)	0.79 (0.62–1.00)	0.74 (0.59–0.93)	0.01
	Multivariable-adjusted ^3^	0.91 (0.84–0.99)	0.03	1.00 (Referent)	0.81 (0.64–1.04)	0.78 (0.61–0.99)	0.04

^1^ Hazard ratios (95% CI) for falls-related hospitalization and fractures by vegetable diversity intakes analyzed using Cox proportional hazard models. ^2^ Test for trend conducted using median value for each vegetable variety category (3, 4, and 5 number/d); ^3^ Multivariable-adjusted model included age, BMI, treatment code, prevalent diabetes mellitus, socioeconomic status, physical activity, smoking history, and energy, protein, calcium, and alcohol intake.

## Supplementary Materials

The following are available online at http://www.mdpi.com/2072-6643/10/8/1081/s1, Figure S1: Percentage distribution of vegetable types by categories of total vegetable intake. Table S1: Mean ± SD grams per day of different types of vegetables, total vegetable intake, and median (IQR) of the variety of vegetables consumed daily based on total vegetable consumption categories.
