# Supplementary material for: Vegetable Diversity, Injurious Falls, and Fracture Risk in Older Women: A Prospective Cohort Study
Source: Nutrients. 2018 Aug 13;10(8):1081. doi: 10.3390/nu10081081 (PMC6115713; doi:10.3390/nu10081081)
Supplement: Supplementary file 1 [file nutrients-10-01081-s001.pdf]

**Supplementary Table 1:** Mean ± SD grams per day of different types of vegetables, total vegetable intake and median (IQR) of the variety of vegetables consumed daily based on total vegetable consumption categories.

| | Categories of Total Vegetable Intake[1] | | |
|---|---|---|---|
| | **<2.2 s/d** | **2.2 to <2.9 s/d** | **≥2.9 s/d** |
| Cruciferous (g/d) | 19.5 ± 13.3 | 31.6 ± 18.3 | 45.2 ± 24.3 |
| Allium (g/d) | 4.9 ± 4.3 | 7.3 ± 5.7 | 11.3 ± 8.2 |
| Yellow/orange/red (g/d) | 31.1 ± 15.3 | 50.8 ± 18.4 | 73.6 ± 28.1 |
| Leafy green (g/d) | 13.5 ± 9.3 | 19.1 ± 11.0 | 23.4 ± 13.3 |
| Legumes (g/d) | 17.7 ± 10.7 | 27.9 ± 15.7 | 36.9 ± 22.3 |
| Others (g/d) | 30.1 ± 16.9 | 52.4 ± 23.5 | 93.6 ± 45.1 |
| Total vegetable intake (g/d) | 116.8 ± 31.4 | 189.2 ± 17.4 | 284.1 ± 59.2 |
| Median (IQR) daily vegetable diversity (number/d) | 3 (2-3) | 4 (4-5) | 5 (4-6) |

[1]Categories were based on 75 g serves per day (s/d) of total vegetable intake; low <2.2 s/d (n=476); moderate: 2.2 to <2.9 s/d (n=477); high: ≥2.9 s/d (n=476). Vegetables were grouped into six types and based on the 2013 Australian Dietary Guidelines and modified slightly to include cruciferous vegetables (cabbage, Brussel sprouts, cauliflower, and broccoli); allium vegetables (onion, leek, and garlic); yellow/orange/red vegetables (tomato, capsicum, beetroot, carrot, and pumpkin); leafy green vegetables (lettuce and other salad greens, celery, silverbeet, and spinach); and legumes (peas, green beans, bean sprouts and alfalfa sprouts, baked beans, soy beans, soy bean curd and tofu, and other beans); and other vegetables (potatoes cooked without fat, mushrooms, cucumber and zucchini).

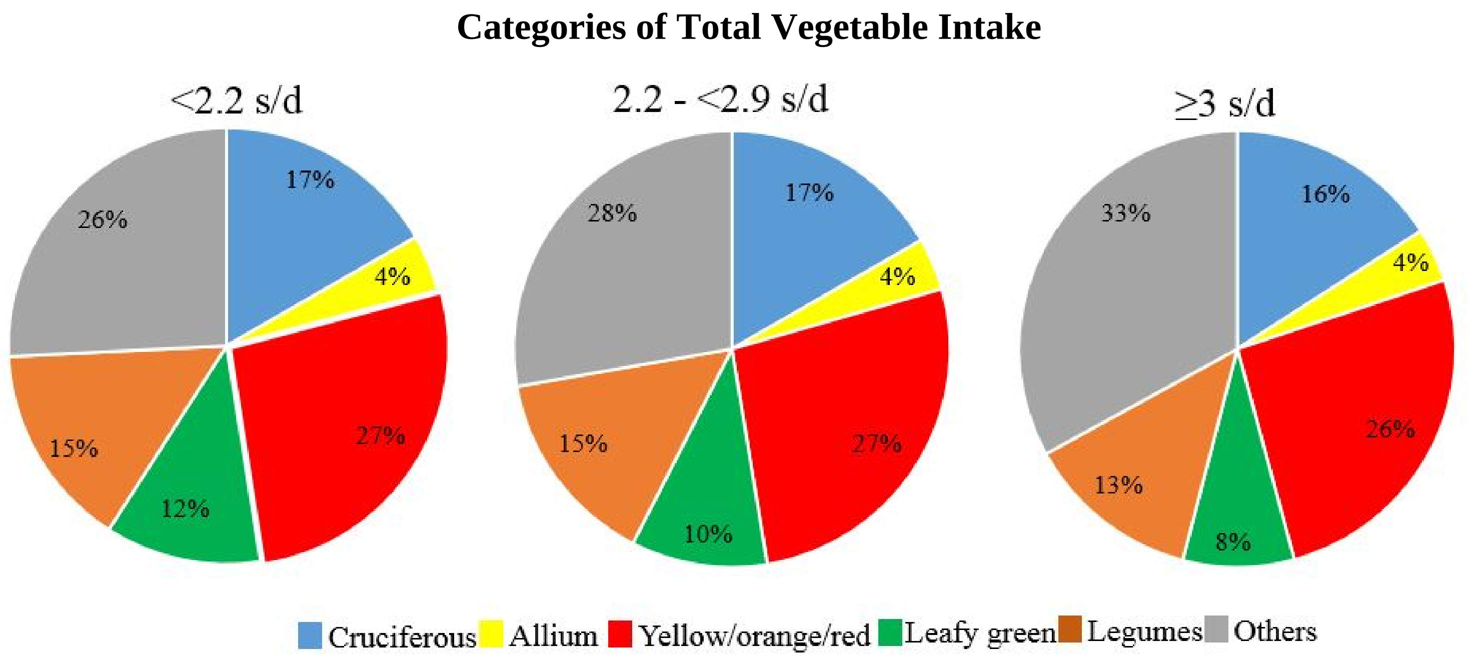


**Supplementary Figure 1:** Percentage distribution of vegetable types by categories of total vegetable intake. Categories were based on 75 g serves per day (s/d) of total vegetable intake; low <2.2 s/d (n=476); moderate: 2.2 to <2.9 s/d (n=477); high: ≥2.9 s/d (n=476). Vegetables types included cruciferous vegetables (cabbage, Brussel sprouts, cauliflower, and broccoli); allium vegetables (onion, leek, and garlic); yellow/orange/red vegetables (tomato, capsicum, beetroot, carrot, and pumpkin); leafy green vegetables (lettuce and other salad greens, celery, silverbeet, and spinach); and legumes (peas, green beans, bean sprouts and alfalfa sprouts, baked beans, soy beans, soy bean curd and tofu, and other beans) and other vegetables (potatoes cooked without fat, mushrooms, cucumber and zucchini).
